# BIBSNet: A Deep Learning Baby Image Brain Segmentation Network for MRI Scans

**DOI:** 10.1101/2023.03.22.533696

**Published:** 2023-05-03

**Authors:** Timothy J. Hendrickson, Paul Reiners, Lucille A. Moore, Anders J. Perrone, Dimitrios Alexopoulos, Erik G. Lee, Martin Styner, Omid Kardan, Taylor A. Chamberlain, Anurima Mummaneni, Henrique A. Caldas, Brad Bower, Sally Stoyell, Tabitha Martin, Sooyeon Sung, Ermias Fair, Jonathan Uriarte-Lopez, Amanda R. Rueter, Essa Yacoub, Monica D. Rosenberg, Christopher D. Smyser, Jed T. Elison, Alice Graham, Damien A. Fair, Eric Feczko

**Affiliations:** 1 Minnesota Supercomputing Institute, University of Minnesota; 2 Masonic Institute for the Developing Brain, University of Minnesota; 3 Washington University; 4 Department of Psychiatry, University of North Carolina at Chapel Hill; 5 Department of Psychology, University of Chicago; 6 University of Michigan; 7 PrimeNeuro; 8 Oregon Health & Science University; 9 Medical School Research Office, University of Minnesota; 10 Institute of Child Development, University of Minnesota; 11 Department of Pediatrics, University of Minnesota; 12 Department of Radiology, University of Minnesota; 13 Center for Magnetic Resonance Research, University of Minnesota

## Abstract

**Objectives:**

Brain segmentation of infant magnetic resonance (MR) images is vitally important in studying developmental mental health and disease. The infant brain undergoes many changes throughout the first years of postnatal life, making tissue segmentation difficult for most existing algorithms. Here, we introduce a deep neural network BIBSNet (**B**aby and **I**nfant **B**rain **S**egmentation Neural **Net**work), an open-source, community-driven model that relies on data augmentation and a large sample size of manually annotated images to facilitate the production of robust and generalizable brain segmentations.

**Experimental Design:**

Included in model training and testing were MR brain images on 84 participants with an age range of 0-8 months (median postmenstrual ages of 13.57 months). Using manually annotated real and synthetic segmentation images, the model was trained using a 10-fold cross-validation procedure. Testing occurred on MRI data processed with the DCAN labs infant-ABCD-BIDS processing pipeline using segmentations produced from gold standard manual annotation, joint-label fusion (JLF), and BIBSNet to assess model performance.

**Principal Observations:**

Using group analyses, results suggest that cortical metrics produced using BIBSNet segmentations outperforms JLF segmentations. Additionally, when analyzing individual differences, BIBSNet segmentations perform even better.

**Conclusions:**

BIBSNet segmentation shows marked improvement over JLF segmentations across all age groups analyzed. The BIBSNet model is 600x faster compared to JLF and can be easily included in other processing pipelines.

## Introduction:

### Infant brain development plays an emerging role in studying mental health and disease

The first years of postnatal life are marked by significant neuronal development through synapse growth, the insulation of neurons (known as myelination), and programmed cell death ^1^. With all of these concurrent processes, this period is particularly dynamic and critical for brain development. Converging evidence suggests that neurodevelopmental and psychiatric disorders later in life are influenced by atypical brain development during this period ^2-6^. Such evidence prompted the NIH to sponsor the Healthy Brain and Child Development (HBCD) study^™^; a large (N > 9,000) multi-site effort to collect infant brain imaging data to study typical and atypical brain development, the effects of in utero substance exposures, and mental health trajectories.

### Infant brain imaging relies on quality brain segmentations

Magnetic resonance imaging (MRI) is a non-invasive methodology for brain imaging and can be used to study developmental brain health and disease. The HBCD study intends to collect multiple types of MR data — diffusion/spectroscopy/functional/quantitative. To perform brain MRI analyses the vast majority of analysis strategies, across many MRI modalities, depend on well-annotated, or segmented, MRI data. One way to segment an infant’s brain is to use structural MR images and create annotations that segment tissue types, such as white matter (WM), gray matter (GM), cerebrospinal fluid (CSF), and subcortical structures^7^. The volumes of segmented regions are important biomarkers on their own ^8^, but accurate segmentation of cortical gray matter is also necessary to produce more advanced morphological metrics such as cortical thickness, surface area, and gyrification ^9^. Finally, other modalities such as fMRI and dMRI rely on accurate segmentations to produce more computationally sophisticated metrics like functional or structural connectivity ^10-14^.

### Automated infant segmentation remains difficult because T1 relaxation contrast varies with infant development

Automated brain segmentation algorithms often rely on high resolution T_1_-weighted (T1w) or T_2_-weighted (T2w) anatomical images to annotate tissue types. These algorithms depend on voxel contrast and intensity differences across differing brain tissue and regions, to delineate brain tissue and region boundaries. Brain tissue and region boundaries are, for the most part, easily delineated within the adult and child brain; however, they are often less accurate in infant data. This is likely due to the significant changes that the brain undergoes during the first years of postnatal life, such as myelination, synaptogenesis, and glial proliferation^1,15,16^. For example, GM and WM voxel contrast is inverted from adults in infants 0 to 3-months (Figure 2), reduced contrast from about 5-9 months causing the tissues to look very similar (Figure 2), and at the later stages of the 5-9 months and on, brains begin to emulate the tissue contrast of an adult brain ^7,17,18^.

### Many current infant brain segmentation methods show limitations when considered for large-scale infant development studies

Most previous literature has focused on designing algorithms that require the input of manually traced brain segmentation atlases based on an infant's age ^19,20^. One popular approach in the field is to register multiple atlases to the image and minimize labeling error with joint label fusion (JLF) ^21,22^. While this strategy has shown to be successful compared to single atlas approaches, the approach is time consuming (computation of 2-3 days) and still error-prone. Often, there is no gold standard method for infant brain segmentation and different strategies may have varying degrees of success for the same age across different scanning sites. As a result, such methods may take months to optimize for a large multi-site, multi-platform study like HBCD. With these limitations in mind, exploring alternative solutions is warranted.

### Deep learning approaches are preferable for large-scale studies, but current implementations utilize limited “ground truth” sample sizes and require skull stripping

Convolutional Neural Networks (CNN) are an attractive alternative, having yielded premier performance with infant brain segmentation ^18,23-26,27^. CNNs provide a scalable solution for multi-site studies, making them ideal for HBCD. CNNs are fast and take only minutes to segment an infant’s brain. CNNs can be further trained with new data from other sites in order to boost generalizability quickly. Table 1 showcases a review of the deep learning infant brain segmentation literature along with limitations of deep learning algorithms. Each study reviewed has limited sample sizes of high-quality ground truth segmentations, with no study exceeding more than 25 infants. Additionally, most studies focus on limited age ranges, specifically between 5-9 months, limiting the generalizability of these models. Next, except for Moeskops et al ^28^, all studies within Table 1 removed the skull, cerebellum, and brain stem from the images prior to segmentation generation. This preprocessing likely reduces the generalizability for images in which the skull remains and increases the burden for performing inference. Finally, based on available models, implementing a model that is open-source, community-centered and follows FAIR principles ^29^ was a vital feature in order to avoid the replication crisis of the 20th century in MRI research. For example, iBEAT ^7^, an infant brain processing and tissue segmentation pipeline, yields excellent tissue segmentation performance (Table 1), however, it is a closed-source software so it is extremely difficult to ascertain the exact methods employed. Additionally, iBEAT can only be executed through a web server or docker, potentially limiting its scalability ^30^.

### Training a deep neural network via nnU-Net and SynthSeg with a large training dataset to accurately and quickly segment 0 to 8-month infant MRI brains

We introduce BIBSNet (**B**aby and **I**nfant **B**rain **S**egmentation Neural **Net**work). BIBSNet was implemented to add to and improve upon the previous deep learning infant and baby brain segmentation research by creating a model that can handle variability of infant MR images based on age, neuronal developmental status, and data acquisition and quality. First, BIBSNet uses a larger sample size, n=84, than has been previously published and employs a relatively wide age range — 0-8 months — with closely sized samples per age range (1-month increments). This wide age range allows BIBSNet to capably segment infant and baby brains across important brain developmental boundaries (i.e,. 0-6 months, and 6-8 months). In particular, the sequences used here are similar to the sequences proposed for the HBCD study, making BIBSNet a suitable starting tool for HBCD. Second, BIBSNet was built upon the proven deep learning architecture, nnU-Net ^31^, for the brain segmentations. Additionally, to make the model more robust to different acquisition types and acquisition idiosyncrasies, SynthSeg was used to create augmented images ^32^. Third, unlike previous literature, BIBSNet does not require that the skull, brain stem, or cerebellum be removed before segmentation, thus requiring much less preprocessing before inference. Lastly, the segmentations produced from BIBSNet are BIDS compatible and in the subject’s native space. This was intentionally done so that BIBSNet segmentations can easily be transformed to fit whatever space that is required for processing and analysis ^33^. Finally, it can be directly input into a preprocessing pipeline, such as NiBabies ^34^ and the DCAN (Developmental Cognition and Neuroimaging) labs infant processing pipeline^10,11,35,36^, making it a turn-key solution for subsequent analyses.

## Methods:

### We used MR images and manually annotated segmentations from infants aged 0 to 8 months from the BCP and ALBERTS datasets for the present study.

#### MR Image Collection Procedure

MR images were collected from 64 participants within the BCP study ^37^ — median postmenstrual age = 64.14 weeks, 40 female — with a 3T Siemens Prisma at the University of Minnesota’s Center for Magnetic Resonance Research (CMRR). The relevant images collected for this study included a T1-weighted (echo time = 2.24 ms, repetition time = 2400 ms, sagittal slices = 208, flip angle = 8°, matrix = 320 x 320, voxel sizes = 0.8 x 0.8 x 0.8mm^3^), a T2-weighted image (echo time = 564 ms, repetition time = 3200 ms, sagittal slices = 208, flip angle = variable, matrix = 320 x 320, voxel sizes = 0.8 x 0.8 x 0.8mm^3^) and approximately 12 minutes of resting state MR images (echo time = 37 ms, repetition time = 800 ms, axial slices = 72, flip angle = 52, matrix = 104 x 91, voxel sizes = 2.0 x 2.0 x 2.0mm^3^) made up of two separate collections with reverse phase encoding (AP and PA).

To supplement the sample with additional neonates, the 20 infants that were used to generate the ALBERTS neonatal atlas were also used ^38^. The MRI data for the ALBERTS infants (median postmenstrual age = 40.855 weeks, 10 female) was acquired on a 3.0 T Philips Intera scanner (Philips Medical Systems, Best, Netherlands). The relevant images collected included T1-weighted (echo time = 4.6ms, repetition time = 17 ms, sagittal slices = 124–150, flip angle = 30°, matrix = 256 × 256, voxel sizes = 0.82 × 0.82 × 1.6 mm^3^) and T2-weighted images (echo time = 160ms, repetition time = 8000 ms, sagittal slices = 88–100, flip angle = 90°, matrix = 224 × 224, voxel sizes = 0.86 × 0.86 × 2.0 mm^3^).

#### Manually Annotated Segmentation Procedure

Following data collection, the T1w and T2w MR images from the BCP study were used to produce manual annotations, based on FreeSurfer’s aseg atlas ^39^. As a starting point for the segmentations, the T1w and T2w images from each BCP participant were run through either JLF or a prototyped version of the BIBSnet algorithm. Led by an expert (E.F.), the cortical structures (GM and WM) from the produced segmentations were then extensively manually annotated with highly trained staff to generate accurate segmentations. Subcortical structures were not specifically annotated except for when they bordered cortical structures. The ALBERTS MR images were included in the training to have fairly equal sample sizes of infants from 0- to 8-months. The segmentations produced from the ALBERTS MR images had been previously manually annotated but did not have extensive quality control procedures and were not reviewed by an expert.

The BCP and ALBERTS MR images and manual annotated segmentations were used for BIBSNet model training and subsequent data processing and analyses.

### BIBSNet combines the nnU-Net model and SynthSeg software, to produce generalizable segmentations

#### nnU-Net model design

**Figure 1 ∣:**
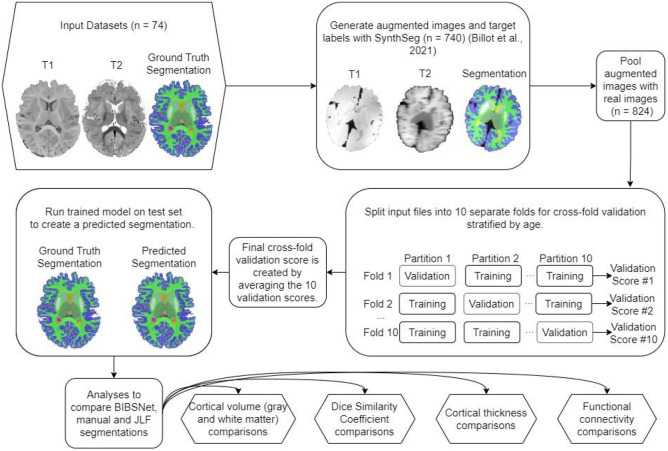
Schematic of model training, validation, and evaluation process. Schematic overview of BIBSNet model design, training, and validation process. Sections shaped as hexagons represent the starting and end points of the current work, whereas sections shaped as rounded rectangles represent intermediate steps.

As can be seen within Figure 1, nnU-Net plays a central role in the BIBSNet model design. nnU-Net is a model that is based upon the CNN based network, U-Net ^40^. In the last several years, U-Net has shown state-of-the-art performance in segmentation problems, specifically biomedical image segmentation. U-Net consists of two separate elements, the contracting path, and the expanding path. The contracting path progressively downsamples the inputted image/s while gaining progressively more in-depth features, known as feature maps, to represent the image. Following the contracting path, the expansive path takes the feature maps and progressively up-samples them until they are the same size as the originally inputted image/s. The downsampling and upsampling is what gives this model its characteristic “U” shape, hence the “U” in U-Net. Despite the success of U-Net, a major pitfall was its difficulty with applying it to different image analysis problems. It required a multitude of expert decisions in the form of adaptation and modification of parameters to be made without clear guidance or obvious parameter defaults. nnU-Net sought to correct this by, 1) fixing parameters that do not require adaptation, 2) tuning parameters that needed to be adapted based upon the inputted dataset; and 3) for any parameters that remain, those decisions would be decided empirically from the data ^31^.

#### SynthSeg

While nnU-Net plays the integral role of segmenting the inputted images, SynthSeg is vital to improve the generalizability of the model. SynthSeg is a software that is capable of producing synthetic MR imaging data. Here, the manually annotated segmentations from the BCP and ALBERTS were inputted into SynthSeg for the production of synthetic MR images. SynthSeg randomly modified the features of each image in four separate ways to emulate different acquisition parameters and data quality ^32^. First, an affine and non-linear transformation was applied to spatially deform the data, simulating a head tilt or changes in head size. Second and third, a randomized Gaussian mixed model and bias corruption were applied. These steps are meant to mimic the global and local pixel intensity differences that can occur across different MRI scanners. Fourth, to mirror acquisition differences resulting from slice thickness, collection orientation, or voxel size the image was randomly downsampled and blurred. For the current study, 740 T1w, T2w, and segmentation datasets were produced based on the 84 participant datasets, a roughly 9x increase in training data. Henceforth, images from the BCP and ALBERTS will be referred to as “real images”, while the synthetic images produced by Synthseg will be referred to as “synthetic images”.

#### BIBSNet validation and training

As shown in Figure 1 the synthetic outputs from SynthSeg, in combination with the real BCP and ALBERTS MR imaging data were fed directly into nnU-Net for segmentation. The same BCP and ALBERT participants were used for training and analysis, thus, cross validation was employed to avoid data leakage. The first step included splitting the real images into ten approximately equally sized pseudorandom testing datasets stratified by age. Each of the ten testing datasets was paired with a training dataset. The training dataset was composed of the other real images along with all generated synthetic images. This strategy ensured that when inference was performed the real images were previously unseen by at least one fold.

To avoid chirality errors, where the inferred annotation was locally correct but the hemisphere was not (e.g. right white matter labeled on the left hemisphere) BIBSnet segmentation outputs underwent a chirality correction procedure. The anatomical T1 was non-linearly registered via ANTS SyN algorithm ^41^ to a template infant T1 of the same age. The template infant comprised a mask of the left and right hemisphere mapped to the T1. The non-linear warp was inversely applied to the left/right mask using a nearest neighbor interpolation, producing the left/right mask in the BIBSnet segmentation space. The mask was then applied to the BIBSnet segmentation, correcting any observed chirality errors.

#### MRI data was processed with the DCAN labs infant-ABCD-BIDS processing pipeline using segmentations produced from manual annotations, and the JLF and BIBSNet models

To test for the effect of segmentation type in subsequent analyses, each of the 84 participants were processed through the DCAN labs infant processing pipeline ^10,35,36,42^ three separate ways: 1) running JLF using the age matched templates produced from the manually annotated segmentations 2) using the externally generated segmentations based on the manual annotations; and 3) using the externally generated segmentation inferred by the trained BIBSNet model. From here on the processing strategies will be referred to as “JLF”, “ground truth” and “BIBSNet”, respectively. Besides the three separate processing strategies mentioned above, the data was processed exactly the same.

The DCAN labs infant processing pipeline is based on the Human Connectome Project minimal processing pipeline ^10^ with key changes to accomodate for the differences in the developing brain of infants. Additionally, the design of DCAN labs infant processing pipeline draws heavily on ABCD-BIDS processing pipeline to accomodate and attenuate scanner effects found within major MRI vendors GE, Philips, and Siemens ^12^. The pipeline is split into several stages: “PreFreeSurfer”, “FreeSurfer”, “PostFreeSurfer”, “FMRIVolume”, “FMRISurface”, “DCANBOLDProcessing”, and “ExecutiveSummary”.

The “PreFreeSurfer” stage, the first stage in the structural pipeline, aligned the T1-weighted and T2-weighted anatomical images, registered native structural images to standard MNI space, and, in the case where of JLF processing, running JLF. The second stage, “FreeSurfer”, ran the software tool FreeSurfer ^9,39,43-47^. The primary goals of FreeSurfer are to reconstruct white and pial cortical surfaces, segment subcortical structures, and to register produced native surfaces to the template space, fsaverage. The final stage in the anatomical structural pipeline is “PostFreeSurfer”. “PostFreeSurfer”, generated the NIFTI volumes and GIFTI surface files and placed them together into CIFTI files for viewing in the visualization tool Connectome Workbench ^10^.

The main goals of the “FMRIVolume" stage, the first stage in the functional pipeline, are to remove spatial distortion, realign volumes to correct for participant motion, register the functional to the structural data, reduce bias field, normalize the resulting 4D image to a global mean, and mask the data ^10,12^. The second functional pipeline stage is “FMRISurface”. The purpose of “FMRISurface" is to extract the time series processed within the volume space and place it into the surface CIFTI standard space. Voxels on the cortical GM ribbon were mapped onto the native cortical surface, transformed according to the surface registration onto the 32k mesh, and finally map the subcortical GM voxels from the set of pre-defined subcortical parcels to a standard set of voxels in each atlas parcel ^10,12^. For infant data, the standard adult MNI template cannot be non-linearly registered for subcortical data. Therefore, here a piecemeal approach was used, where we register each subcortical region linearly to the adult template and then project the subcortical fMRI data into the CIFTI subcortical greyordinates.

Immediately following “FMRISurface” two additional stages “DCANBOLDProcessing”, and “ExecutiveSummary” were launched. The primary goals of “DCANBOLDProcessing” are to further process fMRI CIFTI data by filtering motion estimates by separating true head motion from factitious motion due to magnetic field changes due to breathing^11^, and to produce both dense (dtseries) and parcellated (ptseries) CIFTI files for subsequent analyses. The final stage in the DCAN labs infant processing pipeline, “ExecutiveSummary”, summarized standard quality control outputs and provided them in a browser-interface for easy navigation and review ^12^. Data processing failed for a total of 5 participants despite several attempts. Two participants were ALBERTS and three were from the BCP study. All subsequent analyses excluded these participants.

### Segmentation overlap, and structural and functional MRI metrics were used to perform statistical analyses comparing JLF versus ground truth segmentations, and separately, BIBSNet versus ground truth segmentations

#### Segmentation similarity analysis

To evaluate how similar the segmentations produced by JLF and BIBSNet were to the ground truth segmentations, one metric was used, Dice Similarity Coefficient (DSC) ^48,49^. DSC measures the fraction of overlap between anatomical regions of the same type, ranging in values between 0 and 1. DSC was calculated twice, once to compare JLF and ground truth and, second, comparing BIBSNet and ground truth. DSC is bounded, especially upper bounded so it was assumed that DSC distributions were non-parametric. Therefore, a Wilcoxon signed-rank (WSR) test was used for group comparisons and Cliff’s Delta was used to calculate effect sizes.

#### Structural MRI analyses

In addition to evaluating the similarity of resulting segmentations, morphological metrics were also analyzed. Using outputs from the FreeSurfer processing stage, two different morphological metrics – GM and WM volume – and one cortical sheet metric – average cortical thickness (CT) – were analyzed.

To assess method effectiveness, the metrics produced from BIBSNet segmentations were directly compared using paired T-tests, Cohen’s d, and Normalized Root Mean Squared Error (NRMSE) to the ground truth segmentation produced metrics. Separately, the metrics produced from the JLF model were directly compared to ground truth with the same strategy.

#### Functional MRI analysis

In addition to structural or structurally derived metrics, functional MRI metrics were also analyzed. The Gordon^50^ cortical parcellated resting state time series outputted by the DCANBOLDProcessing processing stage were used to produce pair-pair correlation matrices with the tool “cifti-connectivity” (https://github.com/DCAN-Labs/cifti-connectivity). First, “cifti-connectivity”, used the Gordon cortical parcellated resting state time series processed through the DCAN labs infant processing pipeline. Second, timepoints were regressed out that exceeded a framewise displacement of 0.3mm ^51^. Regressing out time points in this fashion had the effect of removing sufficiently large motion events that could cause spurious correlation. With these motion events removed, each so-called “filtered” Gordon parcellated resting state time series was temporally correlated with itself to produce a pair-pair correlation matrix, also known as a functional connectome matrix. After a preliminary review of available functional connectome data, it was discovered that 3 processed outputs (2 from JLF, 1 from ground truth) had missing values corresponding to regions correlating with the “DIENCEPHALON VENTRAL LEFT" parcel. These missing values were treated as zeroes and used for subsequent analyses.

Like all other metrics, the functional connectome matrices were produced based on each of the three processing strategies. Spearman’s rank correlation was calculated per participant between the ground truth and BIBSNet and, separately, ground truth and JLF. This created a measure of per participant similarity between the JLF and ground truth, and BIBSNet and ground truth functional connectome matrices. Spearman’s rank correlation ranges from 0.0, meaning absolutely no similarity, and 1.0 meaning perfect rank similarity. Like DSC, Spearman's rank correlation is bounded, especially upper bounded, so the values were assumed to be non-parametric. Therefore, a Wilcoxon signed-rank (WSR) test and Cliff’s Delta were used for comparisons. Like the morphological metrics NRMSE was also calculated.

## Results:

### BIBSNet training requires considerable time and resources

The trained BIBSNet model distributed with this work is labeled as “task 512”. Details for downloading and usage can be found on Zenodo and GitHub ^52^. It took approximately 3.5 days of compute time with 1 NVIDIA V100 GPU, 6 CPUs and 90GB of RAM. In practice, running on the Minnesota Supercomputing Institute (MSI) with average delays the total clock time tends to be 5-7 days for training. The training included 5 folds with 1000 epochs per fold. Each epoch takes around four to five minutes to complete. After an initialization step completed within the first fold, all five folds could be run concurrently.

### BIBSNet application is fast and requires minimal resources

Once trained, minimal resources are required to perform inference for an unseen T1w/T2w image pair. The inferences for the present study only required 4 minutes using 2 CPUs, 1 A100 NVIDIA GPU, and 20GB of RAM on the MSI systems. Importantly, a GPU is required to run BIBSNet inference.

Results reveal that ground truth and BIBSNet segmentations are more similar than JLF segmentations based on similarity metrics, and volumetric and cortical brain metrics

### Gray matter volume comparisons

At a group level, differences between BIBSNet versus ground truth and JLF versus ground truth were seen with cortical morphological metrics. As shown in Figure 3a,c (left panels), GM volume produced from BIBSNet segmentation were more similar to ground truth (T = 1.50, p = 0.13, Cohen’s d = 0.05, n = 78) than JLF (T = 6.42, p < 1 x 10^−7^, Cohen’s d = 0.31, n = 78). At an individual level, the NRMSE between JLF and ground truth was 0.17. The NRMSE between BIBSNet and ground truth was 0.09.

### White matter volume comparisons

WM volume comparisons reveal a similar, except more pronounced pattern. As seen within Figure 3a,c (right panels), as a group, ground truth and JLF produced segmentations were substantially different from each other (T = 8.79, p < 1 x 10^−12^, Cohen’s d = 1.10, n = 78), while ground truth and BIBSNet were not (T = 1.81, p = 0.07, Cohen’s d = 0.10, n = 78). Individually the NRMSE between JLF and ground truth was 0.32, and 0.12 between BIBSNet and ground truth.

### Dice similarity coefficient comparisons

DSCs between JLF and ground truth, and BIBSNet and Ground truth were calculated for the GM and WM on the left and right hemispheres, as shown in Figure 3g, Supplemental Figure 1. To be in line with previously conducted morphological and functional metric analyses, DSCs for cortical GM and WM were compared. After a preliminary Shapiro-Wilk analysis, each brain region was revealed to be non-parametric so a Wilcoxon signed-rank test was performed for all subsequent analyses.

The DSC results for the GM and WM exhibited a similar pattern to the cortical morphological metric analyses. Mean DSC of GM between BIBSNet and ground truth (DSC = 0.829) segmentations were significantly larger than the mean DSC between JLF and ground truth (DSC = 0.733) (WSR = 275.0, p < 1 x 10^−8^, Cliff’s Delta = 0.50). The DSC for GM was calculated by averaging values from the “Gray Matter” regions, as shown in Figure 3g, Supplemental Figure 1. Similarly, between BIBSNet and ground truth the mean DSC of WM (DSC = 0.843) was greater than the mean DSC JLF and ground truth (DSC = 0.733) (WSR = 222.0, p < 1 x 10^−8^ Cliff’s Delta = 0.59). Mean WM was calculated by averaging values from the left and right “White Matter" regions.

### Cortical sheet derived metric comparisons reveal a similar pattern to the volumetric cortical metric comparisons

#### Average cortical thickness comparisons

Average CT also revealed a similar pattern to the GM and WM results. As shown within Figure 3b,d,f, both JLF and ground truth produced segmentations (T = 15.46, p < 1 x 10^−24^, Cohen’s d = 2.25, n = 78) and BIBSNet and ground truth produced segmentations (T = 3.37, p < 0.005, Cohen’s d = 0.31, n = 78) were significantly different. Individually, when compared to ground truth the NRMSE for JLF and BIBSNet were 0.37 and 0.10, respectively.

#### Functional connectome matrix comparisons

Like the morphological metrics, differences were also found with the functional connectome metrics. A preliminary Shapiro-Wilk analysis performed on the Spearman rank correlation values revealed, as expected, that they were non-parametric so instead of a paired T-test a Wilcoxon signed-rank test was performed instead. As shown pictorially in Figure 3e, the similarity between BIBSNet and ground truth at a group level was greater than the similarity between the JLF and ground truth produced functional connectome matrices (WSR = 216.0, p < 1 x 10^5^, Cohen’s d = 0.90, n = 55). For the individual comparisons, values for the entire functional connectivity matrices were compared between each group. NRMSE between JLF and ground truth was 0.13 and BIBSNet and ground truth was 0.10.

## Discussion:

### At a group and individual level, cortical volumetric and sheet derived metrics produced from ground truth segmentations were more similar to BIBSNet than JLF

**Figure 2 ∣:**
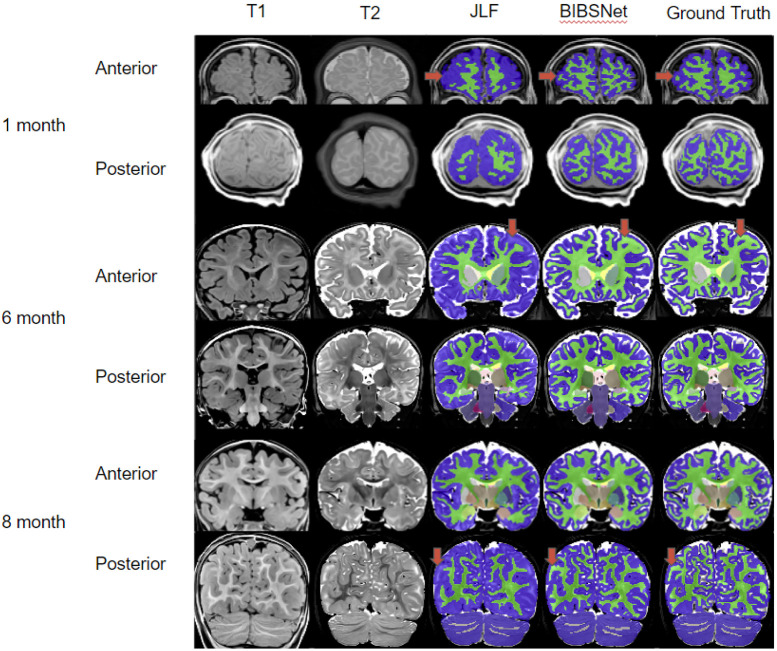
Comparison Pictures between BIBSNet, JLF, and Ground Truth segmentations. Single subject representative slices on the anterior and posterior aspects for 1, 6, and 8 month infants showcasing the T1-weighted, and T2-weighted images, along with the segmentations produced from JLF, BIBSNet, and Ground Truth. The red arrows highlight segmentation label differences. In each case JLF fails to properly segment the white matter – lime green – marking it instead as gray matter – dark blue. Notice that BIBSNet and Ground Truth were able to properly capture the white matter and label it appropriately

**Figure 3 ∣:**
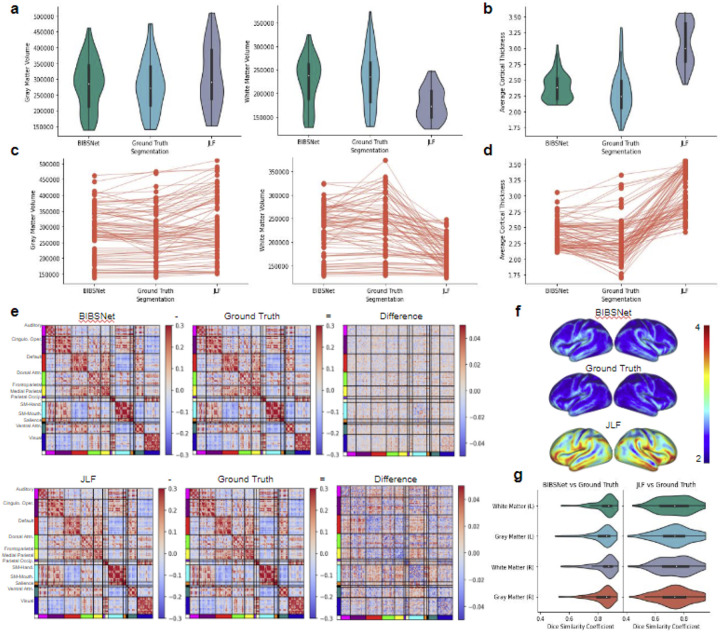
Anatomical and functional derived brain metric comparisons. BCP and ALBERT analysis sample, anatomical data: (**a-d,f,g**, n=78); functional data (**e**, n=55). **a,c**, Gray matter (left panels) and white matter (right panels) volume comparisons based on segmentations produced from BIBSNet, Ground Truth, and JLF. Of note, there is not heavy gray matter volume variability visually between segmentation methods, however, white matter volume certainly does with JLF showing a much lower and tighter distribution compared to Ground Truth. **a**, violin plots – a hybrid of a box and kernel density plot – showcasing the comparisons for each segmentation model grouping, **c**, line plots showcasing the variability in cortical volume by segmentation model used, per participant. **b,d,f**, Cortical thickness values per surface vertex were calculated by FreeSurfer. Cortical thickness values were collapsed to one number by calculating the average per FreeSurfer run (i.e. three surfaces per subject, one for each segmentation model). Visually, much heavier value divergence compared to the cortical volume comparisons. Certainly a palpable difference of BIBSNet and JLF compared to Ground Truth, however, difference is obviously muted for BIBSNet compared to JLF. **b**, violin plots and **d** line plots showcasing the comparisons for each segmentation model grouping. **f**, whole brain cortical thickness visualizations averaged by segmentation grouping (colorbar values are represented by cortical thickness in mm). **e**, Difference maps of segmentation group functional connectivity matrices derived from the Gordon parcellation atlas. BIBSNet (top) and JLF (bottom), separately were subtracted from by the Ground Truth functional connectivity matrix to produce a difference map. Notice that the values of the difference map between BIBSNet and Ground Truth hover more closer to zero (i.e. no difference), compared to the JLF and Ground Truth difference map. **g**, Dice Similarity Coefficient comparisons between BIBSNet and Ground Truth segmentations (left) and JLF and Ground Truth segmentation (right) across the left and right hemisphere gray and white matter volumes. Notice that on average the BIBSNet vs Ground Truth DSC is much higher, compared to JLF vs Ground Truth measures

Across all cortical and cortically derived metrics, BIBSNet was more similar to the ground truth than JLF. DSC (Figure 3g) across all cortical structures shows that BIBSNet (DSC = 0.836) outperformed JLF (DSC = 0.733) by 13%. Additionally, BIBSNet outperformed JLF by 13% and 15% for white matter and gray matter DSC comparisons, respectively. Based on Cliff’s delta these effects were both considered to be large.This effect was confirmed with cortically derived metrics, based on Cohen’s d effect sizes and NRMSE. Interestingly, it appears that JLF and BIBSNet perform similarly to ground truth for the younger and older infants, but JLF shows a performance dip for infants between 3-6 months (Supplemental Figure 1). This likely corresponds to the reduced contrast found in infants during this age range (Figure 2).

Cohen’s d effect sizes represent the difference between two means in standard deviations. They are typically interpreted as follows, d = 0.2 is considered to be a ‘small’ effect, d = 0.5 is a ‘medium’ effect’ and d = 0.8 is a ‘large’ effect. While Cohen’s d represents the size of an effect based on a group, NRMSE represents it individually. NRMSE represents the amount of error in a model by 1) measuring how close values predicted by a model are to observed values (residuals), 2) squaring the residuals, 3) averaging across all residuals 4) taking the square root; and then 5) dividing by the mean of the observed values to normalize.

The mean group white matter volume differences were more than one standard deviation apart (Cohen’s d = 1.10) between JLF and ground truth indicating a very large effect (Figure 3a**, right panels**), but only about 0.1 standard deviations apart between BIBSNet and ground truth, indicating a very small effect. The NRMSE between JLF and ground truth WM volumes were about 2.6x larger than between BIBSNet and ground truth. This does not mean that JLF made 2.6x larger errors than BIBSNet when compared to ground truth; however, predicted values from JLF deviated more heavily from ground truth than BIBSNet and likely made errors at a larger rate. GM volume differences followed the same trend (Figure 3a**, left panels**) both at a group and individual level as WM, but were not as pronounced. This result also is illuminated when visualized pictorially. Notice in Figure 2 that while BIBSNet makes some small error compared to ground truth, JLF mislabels a multitude of WM as GM, as indicated by the arrows.

CT relies on boundaries drawn between WM and GM, and GM and the pial surface for metric calculation ^53,54^. Likely due to this relationship, the findings between JLF and ground truth and BIBSNet and ground truth were even more pronounced for CT (Figure 3b,d,f). The group means between JLF and ground truth differed largely, more than 2 standard deviations while BIBSNet and ground truth mildly to moderately differed from each other. As expected this same pattern was observed individually with NRMSE between JLF and ground truth being more than 3x larger than NRMSE between BIBSNet and ground truth.

Like CT, producing functional connectivity matrices relies on accurately drawn boundaries. With functional analyses boundaries are used to define where rsfMRI voxels fall within the cortical gray matter ribbon ^10^. Thus, it was expected that functional connectivity matrix comparisons would reveal a similar pattern to CT. This was confirmed statistically and visually within Figure 3e. These findings are not unexpected, however, it is reassuring that the success of BIBSNet segmentation also extends to a different MR image modality.

### Results in context of previous literature

The DSC findings within this present work are in line with previous literature (Table 1). Some models certainly out performed BIBSNet, however, those models had such small sample sizes that poor generalizability and overfitting are likely. The morphological and resting state fMRI analyses conducted here is the most extensive within the deep learning infant brain segmentation generation literature that we are aware of. Our hope is that the analyses performed and the screenshots showcased within Figure 2 will allow researchers to see the practical benefits to using BIBSNet. Additionally, our hope is that the ease with which BIBSNet can be deployed by others will lead to widespread adoption within the infant MRI neuroimaging research space.

### Limitations and future directions will extend BIBSNet

The BIBSNet trained model performs well with cortical structures, but it also isn’t without limitations. The primary limitation is that the ground truth annotation of subcortical structures was not a focus. Even though BIBSNet does produce a full FreeSurfer ‘aseg’ segmentation the gold standard ground truth segmentations are incomplete and thus are not yet optimally trained A secondary limitation is that BIBSNet requires a T1w and T2w image pair to work. Often with infant research MR scans, only one of the two images are collected. Since infant MR scans are acquired while the infant is asleep, time is at a premium. Therefore, once a single anatomical image is collected, researchers often move on to collecting other images such as resting state or diffusion weighted A final limitation is that despite the large number of ground truth annotations used for training, the training dataset only consisted of infants 0 to 8-months of age Additionally, the training data was from just two studies, BCP and ALBERTS. The limited age range and number of studies may potentially limit BIBSNet, however, we are cautiously optimistic that use of SynthSeg will boost model generalizability.

Based on these limitations, future work will focus on several different aspects. First, internal processes to improve ground truth annotation of subcortical structures is already underway and will be a large focus in subsequent BIBSNet model releases. Second, building models that will only require only T1-weighted or T2-weighted images is already in process. Third, expanding the model into 12-36-month data, and beyond, and incorporating additional acquisition/sequences is planned for version 2 of the model. Fourth, assessing model performance on synthetically produced T1 and T2 weighted images from quantitative imaging or deep learning ^55^. Finally, applying the BIBSNet model to large and diverse datasets, such as HBCD, to assess model generalizability will be performed.

## Conclusion:

This work examined the performance of the BIBSNet model compared to the existing state-of-the-art JLF algorithm. BIBSNet saliently outperformed JLF across all cortical or cortically derived metrics analyzed. BIBSNet is the only open-source pre-trained deep neural network model that we are aware of to construct high-quality cortical segmentations in baby and infant brains. BIBSNet performed well across multiple ages and was robust to the existence of the skull in images. Finally, the pre-trained model is fast (at least 600x speed up compared to JLF), requires minimal high performance computing resources, and can easily be included in other pipelines.

## Supplementary Material

1

## Figures and Tables

**Table 1 ∣:** Spreadsheet of previous literature.

Authors, Year	Paper Title	Sample Size	Deep Learning Architecture	Anatomy Removed from Image	Dice Similarity Coefficient
Zhang, 2015	Deep convolutional neural networks for multi-modality isointense infant brain image segmentation	8: 26-34 wks	Convolutional Neural Network	skull, cerebellum, brain stem	GM: 0.86, WM: 0.85
Moeskops, 2017	Isointense infant brain MRI segmentation with a dilated convolutional neural network	23: 26 wks	Convolutional Neural Network	skull, cerebellum, brain stem	WM: 0.874, GM: 0.877
Moeskops, 2016	Automatic Segmentation of MR Brain Images with a Convolutional Neural Network	10: 30 wks 12: 40 wks	Convolutional Neural Network	None	30 weeks: CB: 0.92, mWM: 0.69, BGT: 0.92, uWM: 0.96, BS 0.87, cGM: 0.84, 40 weeks: CB: 0.93, mWM: 0.56, BGT: 0.91, uWM: 0.93, BS: 0.85, cGM: 0.87
Nie, 2016	FULLY CONVOLUTIONAL NETWORKS FOR MULTI-MODALITY ISOINTENSE INFANT BRAIN IMAGE SEGMENTATION	10: 26-34 wks	multi fusion fully convoluted network	skull, cerebellum, brain stem	GM: 0.873, WM: 0.887
Dolz, 2018	ISOINTENSE INFANT BRAIN SEGMENTATION WITH A HYPER-DENSE CONNECTED CONVOLUTIONAL NEURAL NETWORK	23: 26 wks.	3D hyper dense CNN known as HyperDenseNet	skull, cerebellum, brain stem	GM: 0.920, WM: 0.901
Dolz, 2020	Deep CNN ensembles and suggestive annotations for infant brain MRI segmentation	23: 26 wks	3D CNN SemiDenseNet, extending HyperDenseNet.	skull, cerebellum, brain stem	GM: 0.92, WM: 0.90
Nie, 2019	3-D Fully Convolutional Networks for Multimodal Isointense Infant Brain Image Segmentation	11: 26-34 wks	3D multimodal fully convoluted network	skull, cerebellum, brain stem	GM: 0.8817, WM: 0.8586
Zeng, 2018	MULTI-STREAM 3D FCN WITH MULTI-SCALE DEEP SUPERVISION FOR MULTI-MODALITY ISOINTENSE INFANT BRAIN MR IMAGE SEGMENTATION	23: 26 wks	3D fully convoluted network	skull, cerebellum, brain stem	GM: 0.916, WM: 0.896
Wang, 2018	Volume-Based Analysis of 6-Month-Old Infant Brain MRI for Autism Biomarker Identification and Early Diagnosis	18: 26 wks	Anatomy-Guided Densely-Connected U-Net	skull	GM: 0.923, WM: 0.933

Sample size, deep learning architectures, anatomy removed and Dice similarity coefficient reported in the recent infant brain segmentation literature. GM: gray matter, WM: white matter, CB: cerebellum, mWM: myelinated white matter, BGT: basal ganglia and thalami, vCSF: ventricular cerebrospinal fluid, uWM: unmyelinated white matter, BS: brain stem, cGM: cortical gray matter, eCSF extracerebral cerebrospinal fluid
