# Supplementary material for: BIBSNet: A Deep Learning Baby Image Brain Segmentation Network for MRI Scans
Source: bioRxiv. 2023 May 3:2023.03.22.533696. Originally published 2023 Mar 24. Preprint. [Version 2] doi: 10.1101/2023.03.22.533696 (PMC10055337; doi:10.1101/2023.03.22.533696)
Supplement: Supplementary file 1 [file NIHPP2023.03.22.533696v2-supplement-1.pdf]

bioRxiv preprint doi: https://doi.org/10.1101/2023.03.22.533696; this version posted May 3, 2023. The copyright holder for this preprint (which was not certified by peer review) is the author/funder, who has granted bioRxiv a license to display the preprint in perpetuity. It is made available under aCC-BY 4.0 International license.

Supplemental Figures:

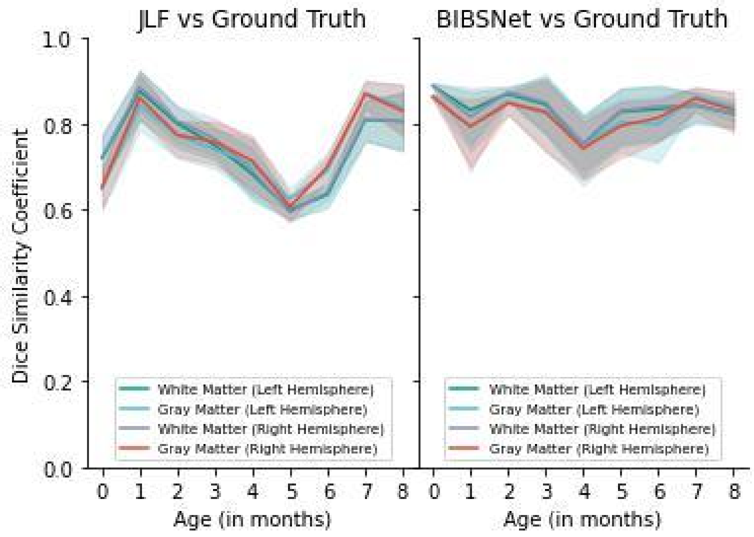

Supplemental Figure 1 | Dice similarity coefficients by infant age full sample.
Line plots outlining the per subject Dice similarity coefficient by infant age (in months) between paired participants (n=78) for JLF and Ground Truth annotated segmentations (left) and BIBSNet and Ground Truth (right) annotated segmentations. Since there are repeat values (age in months) the lineplot aggregates over these showcasing the mean (opaque lines) and 95%

bioRxiv preprint doi: https://doi.org/10.1101/2023.03.22.533696; this version posted May 3, 2023. The copyright holder for this preprint (which was not certified by peer review) is the author/funder, who has granted bioRxiv a license to display the preprint in perpetuity. It is made available under aCC-BY 4.0 International license.

confidence interval (semi-transparent lines). Observe that JLF and BIBSNet seem to perform very similarly to Ground Truth for infants in the 0-1 month and 6-8 month ranges. However, JLF shows a heavy performance drop for infants between 3-6 months of age, whereas BIBSNet still performs relatively equally to Ground Truth over this same range.
